# Anticancer effects of dihydromyricetin on the proliferation, migration, apoptosis and in vivo tumorigenicity of human hepatocellular carcinoma Hep3B cells

**DOI:** 10.1186/s12906-021-03356-5

**Published:** 2021-07-06

**Authors:** Lianggui Jiang, Wen-Chu Ye, Zuobiao Li, Yongguang Yang, Wei Dai, Mingyi Li

**Affiliations:** 1 Laboratory of Hepatobiliary Surgery, Zhanjiang Key Laboratory of Hepatobiliary Diseases, Affiliated Hospital of Guangdong Medical University, Zhanjiang, Guangdong 524001 People’s Republic of China; 2 Department of Thyroid and Breast Surgery, The People’s Hospital of Ganzhou, Ganzhou Affiliated Hospital of Nanchang University, Ganzhou, Jiangxi 341000 P.R. China; 3 The Sixth Affiliated Hospital of Guangzhou Medical University, Qingyuan People’s Hospital, Qingyuan, 511518 Guangdong China

**Keywords:** Hepatocellular carcinoma, Dihydromyricetin, Anticancer effect, Apoptosis, Migration; proliferation

## Abstract

**Background:**

Hepatocellular carcinoma (HCC) represents a serious public health problem worldwide and has high morbidity and mortality. Dihydromyricetin (DHM) exhibits anticancer effect on a variety of malignancies, but its anticancer function of DHM in HCC has been unclear. The aim of this study was designed to investigate the anticancer effect of DHM on cell apoptosis, proliferation, migration and invasion of hepatoma carcinoma cells.

**Methods:**

Cultured Hep3B cells were treated with different DHM concentrations, followed by cell apoptosis, proliferation, migration and invasion were examined by CCK-8, colony formation assay, wound healing, Transwell and flow cytometry, respectively. The mRNA and protein expression of BCL-2, Cleaved-caspase 3, Cleaved-caspase 9, BAK, BAX and BAD were validated by western blot.

**Results:**

DHM markedly suppressed proliferation, migration, invasion and facilitated apoptosis in Hep3B cells. Mechanistically, DHM significantly downregulated the Bcl-2 expression, and upregulated the mRNA and protein levels of Cleaved-Caspase 3, Cleaved- Caspase 9, Bak, Bax and Bad. Furthermore, in the nude mice tumorigenic model, DHM treatment greatly decreased the weight of the HCC cancers compared to the weights in control and NDP group.

**Conclusions:**

DHM could suppress cell proliferation, migration, invasion, and facilitated apoptosis in Hep3B cells. These findings could provide novel insights to develop potential therapeutic strategy for the clinical treatment of HCC.

**Supplementary Information:**

The online version contains supplementary material available at 10.1186/s12906-021-03356-5.

## Introduction

Hepatocellular carcinoma (HCC) is the most common type of primary hepatocellular carcinoma and is a growing public health problem worldwide. Recently, the incidence, recurrence and mortality of HCC are continuously increasing over years in the majority of countries [1]. The increase in mortality mainly due to a lack of effective therapeutic options [2]. However, most treatments may cause serious side effects, such as nephrotoxicity, neurotoxicity and gastrointestinal (GI) toxicity [3]. Recent studies have illustrated that traditional Chinese medicines (TCMs) have benefic effects on the treatment of a variety of cancers, including HCC [4–6]. Therefore, to find a novel and effective TCM for the treatment of HCC, with the aim to improve the overall survival time of patients with HCC.

Dihydromyricetin (DHM), a biologically active flavonoid compound from Ampelopsis grossedentata [7], exerts anti-inflammatory, hypoglycemic, antioxidative, antimicrobial, anti-allergic, and anti-acne effects [8]. Remarkably, this flavonoid compound has attracted considerable attention because of its strong inhibitory effect on colorectal cancer [9], ovarian cancer [10], cholangiocarcinoma, and lung cancer [11]. It has been documented that DHM inhibited the progression of colorectal cancer and colon cancer in mouse models [9, 12]. Besides, DHM also plays an important role in various biological processes including cell proliferation, apoptosis, and migration [13]. In vitro experiment demonstrated that DHM suppressed cell proliferation, migration, invasion and promoted apoptosis and cell cycle arrest at the G1/S phase in melanoma SK-MEL-28 cells, HCC [14], ovarian cancer cells [10], lung cancer cells [15], myelomonocytic lymphoma cells [16], and cholangiocarcinoma cells [13, 17].

In the present study, we mainly investigated the effect of DHM in the biological processes of cell growth and metastasis in Hep3B cells. The different concentrations of DHM were used to treat Hep3B cells to reveal the function of DHM on cell proliferation, migration and apoptosis. DHM treated Hep3B cells to reveal the anticancer characteristics both in vivo *and* in vitro. The apoptosis-associated genes and Bcl-2/Caspase-9 signaling pathway were analyzed. Further, in the nude mice tumorigenic model, DHM treatment significantly reduced the weight of the HCC cancers. These findings might provide a potential therapeutic candidate for the clinical treatment of HCC.

## Materials and methods

### Cell culture and treatment

Hep3B cells derived from American Type Culture Collection (ATCC, Rockville, MD, USA) were grown in Dulbecco’s modified Eagle medium (DMEM, Life Technologies, Carlsbad, CA, USA) with 10% fetal bovine serum (FBS, Gibco, Australia). The cells were incubated in an incubator containing 95% air and 5% CO_2_ at 37 °C. Dihydromyricetin (DHM) obtained from Sigma-Aldrich (St Louis, MO, USA) was dissolved in dimethylsulfoxide (DMSO, Sigma-Aldrich) and then different concentrations of DHM were used to treat Hep3B cells. Nedaplatin (NDP, Nichi-Iko Pharmaceutical Co., Ltd.) was solubilized in sterilized H2O. DMSO served as a control group.

### Animals and tumor models

The mice (4–6 weeks) were housed under standard animal room conditions (temperature 22 ± 1 °C and humidity 55 ± 5%). Mice were anesthetized with 2% isoflurane (via inhalation) (RWD, Shenzhen, China) using a Rodent Anesthesia Machine (VetEquip Inc., Pleasanton, Ca). Animals were sacrificed with 2–3 times the anesthetic dose of isoflurane, followed by cervical dislocation, shaven and sterilized with 75% ethanol [18]. After opening the abdominal cavity, the liver and tumor tissue were collected and weighed. All animals had free access to sterile tap water and food during the experiments. The mice were randomly divided into three groups, including control groups (*n* = 8), DHM (n = 8) and NDP (n = 8) for 3 weeks. Hep3B cells were transplanted into the mouse via subcutaneous injection of 1 × 10^7^ cells [19]. One week after transplantation, tumors had grown to a volume of approximately 20 mm^3^ with a model success rate of 100%. All the animal experiments and surgical procedures were approved by the Institutional Animal Care and Use Committee of Guangdong Medical University (GDY1802018).

### Cell viability measurement

The cell viability was assayed by adding Cell Counting Kit-8 assays (CCK-8) solution as described (CCK-8, Dojindo Molecular Technologies, Gaithersburg, MD) [20, 21]. The Hep3B cells were seeded in 6-well plates (1 × 10^5^ cells/ well) and were allowed to adhere for 8 h. The medium was replaced with medium containing different concentrations of DHM (0, 10, 20, 30, 40, 50 μM). DMSO control wells contained 0.1% DMSO. After 24 h, the culture medium of the cells was discarded, 10 μL of CCK-8 solution was added into each well and cells were incubated at 37 °C for another 2 h. Finally, the absorbance was analyzed at 450 nm using a Microplate Reader (Molecular Devices, San Jose, CA, USA). All the assays were performed for three times independently. Absorbance of cells in the absence of treatment was regarded as 100% of cell survival. Cell survival was calculated as: absorbance/absorbance of control × 100%.

### Colony formation assay

Cell viability was performed using a colony formation assay [22]. Hep3B cells were seeded into a 6-well plate (3 × 10^2^ cells/well) for 8 h, followed by treatment with two different concentrations of DHM and NDP for 24 h. Hep3B cells were cultured with drug-containing medium for ten days. The cells were fixed with methanol-glacial acetic acid stationary solution (3:1) at room temperature for 10 min and stained with 1% crystal violet (Amresco, Solon, OH, USA). The following formula was used to calculate the colony formation inhibition rate: Colony formation inhibition rate = (control group -experimental group)/control group × 100%.

### Cell apoptosis assay

Cell apoptosis was assessed by flow cytometry assay (BD, FranklinLakes, NJ) [23]. In brief, the cells were seeded in 6-well plates (1 × 10^6^ cells/well), followed by 24 h incubation at 37 °C. The cells were then treated with different concentrations of DHM and NDP for 24 h. The assay was performed using the Annexin V-FITC/PI cell apoptosis detection kit (BD Pharmingen, USA) according to the manufacturer’s protocol. Subsequently, the cells were monitored by flow cytometry (FACSCalibur, Becton Dickinson, USA), and the data were analyzed using FlowJo™ software (version 10, FlowJo LLC).

### Cell migration and invasion assay

Cell migration and invasion were detected by using Transwell assay with 8.0 μm porous polycarbonate membranes (Millipore, Bedford, Massachusetts, USA) [21]. In brief, cells were treated with different concentration of DHM and NDP and adjusted cell density to 1 × 10^5^. The lower transwell contained 600 μl DMEM with 10% FBS. After incubation 24 h at 37 °C, the non-traversed cells in the upper compartment were wiped by a wet cotton swab. Traversed cells on the lower side of the filter were fixed stained with 0.1% crystal violet (Amresco, Solon, OH, USA). Then these cells were stained with 0.5% crystal violet (Merck, Darmstadt, Germany) for 20 min and counted microscopically (Olympus, Tokyo, Japan). The method of cell invasion was similar with cell migration, except that the inserts were coated with BD MatrigelTM Matrix (BD Biosciences, NY, USA).

### Wound healing assay

Cell migratory abilities was tested by a wound healing assay. Hep3B cells were seeded in 12-well dishes (5 × 10^4^ cells/well), and incubated in DMEM with 10% FBS for 24 h at 37 °C. The cells were then exposed in the absence or presence of DHM and NDP. Then the cells were scratched across the surface of the well by pipette tip. After an incubation at 37 °C of 24 h, the scratches were observed.

### Western blot analysis

The effects of DHM and NDP on the expression levels of Bcl-2, Cleaved-Caspase 3, Cleaved- Caspase 9, Bak, Bax and Bad were analyzed using western blot [21]. Protein samples were obtained from Hep3B cells that were treated with different concentrations of DHM and NDP for 24 h using cell lysis buffer (RIPA, Beyotime Biotechnology, Shanghai, China). The proteins were collected and detected by using the BCA™ Protein Assay Kit (Pierce, Appleton, WI, USA). Subsequently, total protein (20 μg) samples were separated using SDS-PAGE (10% gel) and transferred onto a polyvinylidene fluoride (PVDF) membranes and blocked in 5% skim milk powder for 1 h at room temperature. Following the membranes were incubated with the corresponding antibodies. Primary antibodies of Bcl-2, Cleaved-Caspase 3, Cleaved- Caspase 9, Bak, Bax and Bad were (antibodies shown in Table 1) were incubated with the membrane at 4 °C overnight. Then, blots were washed three times with TBST and were incubated with secondary anti-bodies for 1 h at room temperature. The blots were detected using enhanced chemiluminescence (ECL) reagents (Super Signal Dura kit, Pierce, IL, USA) according to the manufacturer’s instructions. The blots were quantified by using Image Lab™ Software (Bio-Rad).

**Table 1:** Antibodies used in the study

Antibodies	Manufacturer	Catalogue numbers	Observed MW	Dilution
BCL-2	Abcam	ab32124	26 kDa	1:1000
Cleaved-Caspase 3	Abcam	ab49822	17 kDa	1:500
Cleaved- Caspase 9	Affinity Biosciences	AF5240	10 kDa	1:1000
Caspase 9	Abcam	ab32539	46 kDa	1:1000
BAK	Abcam	ab32371	23 kDa	1:10000
BAX	Abcam	ab32503	21 kDa	1:1000
BAD	Abcam	ab32445	18 kDa	1:2000
GAPDH	absin	abs132004	37 kDa	1:3000

### Hematoxylin and eosin staining

Briefly, liver tissues were immersed in 4% paraformaldehyde for 4 h and transferred to ethanol (75, 85, 95%). Then they were treated with xylene paraffin-embedded according to a previous report [24]. Before immunostaining, 3-μm-thick liver tissue sections were dewaxed in xylene, rehydrated by decreasing concentrations of ethanol (95, 85, 75%) and washed in PBS. Tissue sections were then stained with hematoxylin and eosin (H&E). After staining sections were dehydrated through increasing concentrations of ethanol and xylene.

### Statements

The study was approved by ARRIVE guidelines (http://www.nc3rs.org.uk/arrive-guidelines). Moreover, we confirm that all methods were performed in accordance with the relevant guidelines and regulations.

### Statistical analysis

All data are expressed as the mean ± S.E.M., and at least three independent replicates were used for per group. All statistical procedures were analyzed by SPSS 22.0 (IBM, Manassas, VA, USA), and plots were generated using GraphPad prism 8.0 (GraphPad Software, La Jolla, CA, USA) (https://www.graphpad.com/scientific-software/prism/). The SPSS analysis shows that our results are normal distribution, and homogeneity of results between each treatment groups are equal. Significant differences between treatment groups were determined by one-way ANOVA (SPSS 22.0, Chicago, IL, USA).

## Results

### DHM suppressed cell proliferation and viability of Hep3B cells

**Fig. 1:**
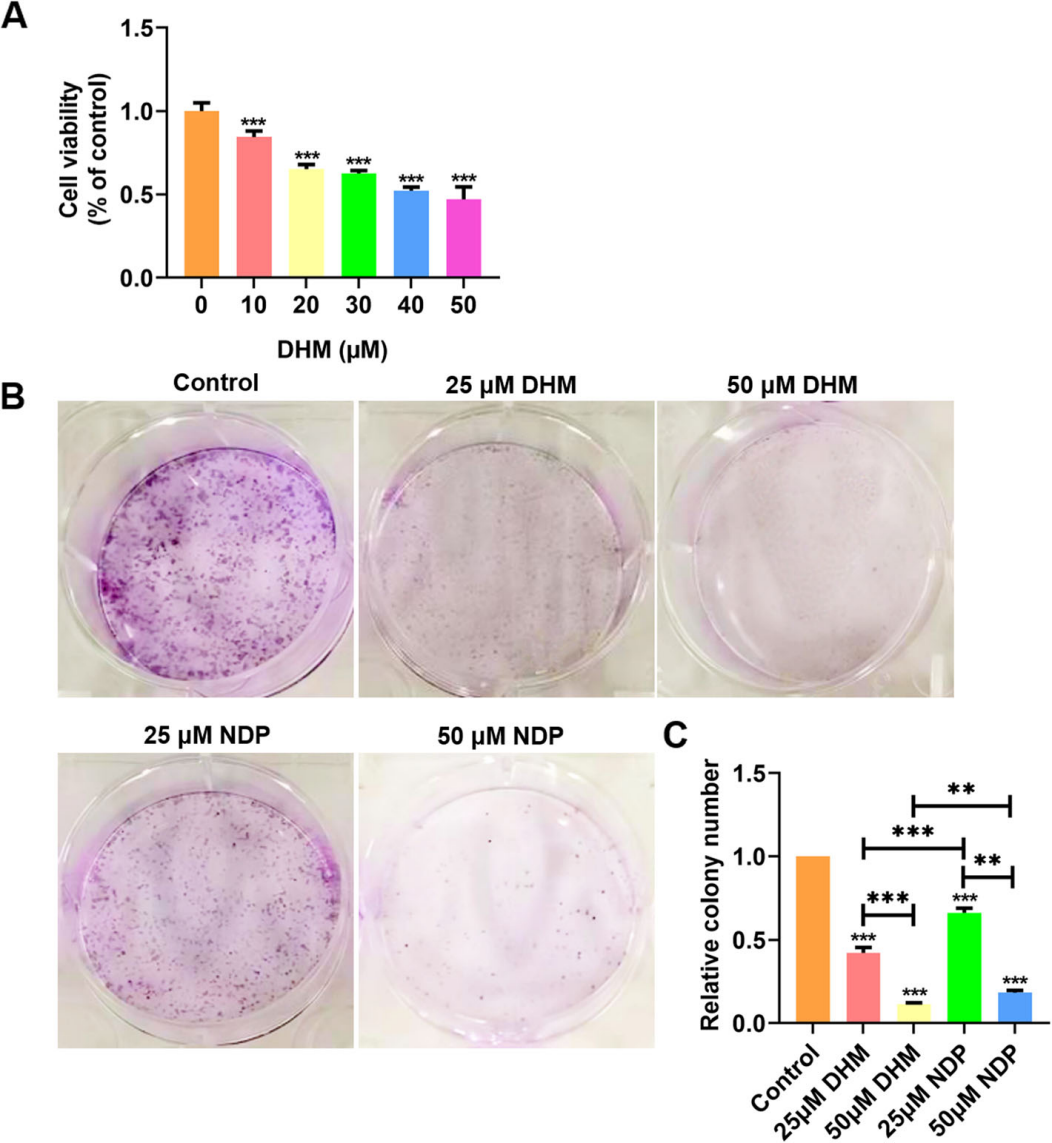
DHM inhibited cell proliferation and viability of Hep3B cells. **A** Hep3B cells were treated with six different doses of DHM (0 μM, 10 μM, 20 μM, 30 μM, 40 μM, 50 μM) of DHM for 24 h. Cell proliferation was examined by CCK-8 assays. **B** Effects of DHM and NDP on viability of Hep3B cells according to a colony formation assay. **C** Quantification of colonies number in the colony formation assay. Results are presented as mean ± standard error of mean (*n* ≥ 3). **P < 0.01 and ***P < 0.001 vs. control group or NDP group. DHM, Dihydromyricetin; NDP, Nedaplatin

The Hep3B cells were cultured with different concentrations of DHM (0 μM, 10 μM, 20 μM, 30 μM, 40 μM, 50 μM for 24 h, and then the cell proliferation was measured by CCK-8 assay. The results of the CCK-8 assay revealed that the proliferation of cells in the DHM group differed compared with the control group (Fig. 1A). The inhibitory effect increased prominently with increasing DHM concentration in a dose-dependent manner (*P* < 0.001) (Fig. 1A). In the subsequent experiments, 25 and 50 μM of DHM were selected to treat Hep3B cells. According to the colony formation assay results (Fig. 1B and C), the colony formation ability in each group was significantly inhibited compared with the blank control group (*p* < 0.001), and the inhibition rate of 25 and 50 μM DHM (57.85 ± 3.24%; 88.55% ± 0.759%) were higher compared with the same NDP (33.64 ± 2.73%; 81.55% ± 1.41%). These data indicated that DHM inhibited the viability and proliferation of Hep3B cells, and its effect was comparable to that of NDP.

### DHM inhibited the cell migration and invasion of Hep3B cells

**Fig. 2:**
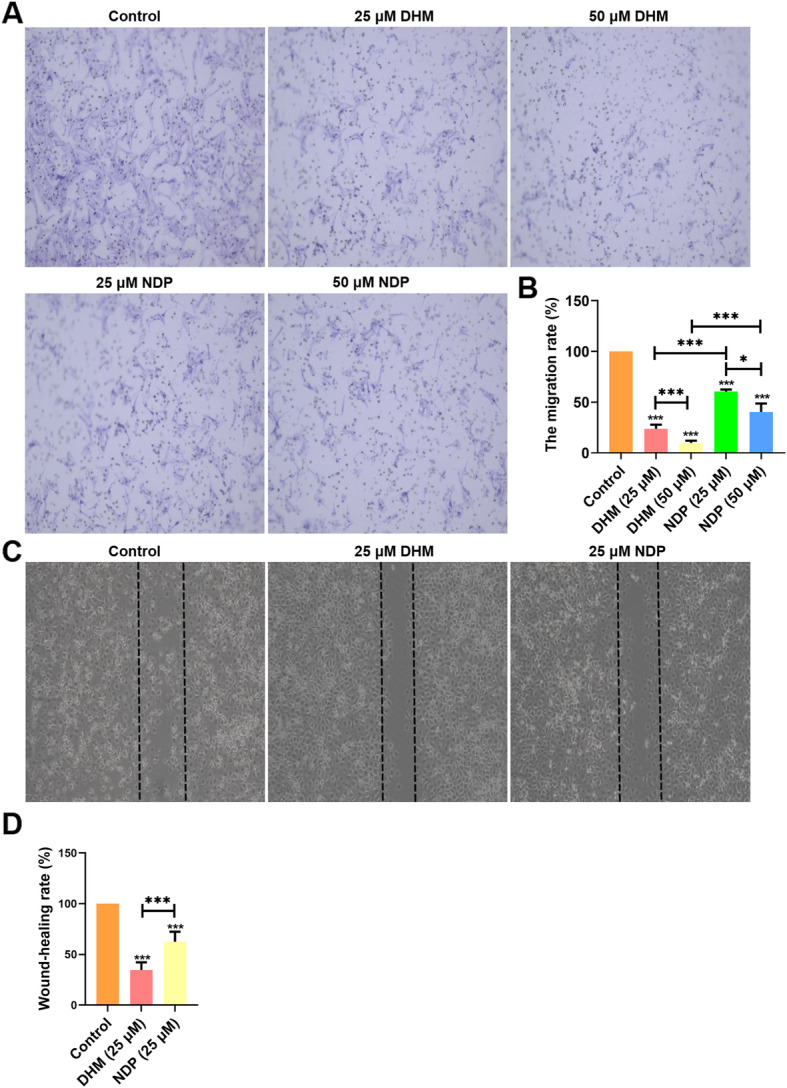
DHM inhibited cell migration and invasion of Hep3B cells. **A** Effect of DHM and NDP on the migratory ability of Hep3B cells in a Transwell assay. **B** Quantification of migration rate in the Transwell assay. Magnification, × 200. ***P < 0.001. **C** Wound-healing assay. **D** Quantification of wound-healing rate in the wound healing assay. Results are presented as mean ± standard error of mean (*n* ≥ 3). **P < 0.01 and ***P < 0.001 vs. control group or NDP group. NDP, Nedaplatin

Cell migration and invasion of Hep3B cells were measured by Transwell assay in this study. The results of Transwell assay (Fig. 2A) illustrated that numerous cells migrated into the membrane of the upper chamber in the control group, whereas different doses of DHM and NDP treatment significantly inhibited the cell migration rate (*P* < 0.001) (Fig. 2A and B). Notably, the inhibitory effect on migration increased gradually with increasing DHM and NDP concentration in a dose-dependent manner (*P* < 0.001). The migration rate of the control group was obviously increased in comparison with that of the treatment group, 23.79 ± 3.97% in the DHM (25 μM), 9.78 ± 2.33% in the DHM (50 μM), 60.42 ± 2.05% in the NDP (25 μM), and 40.26 ± 8.44% in the NDP (50 μM) treatment groups (Fig. 2B). Furthermore, the wound healing assay revealed that DHM and NDP treatment significantly reduced wound closure rates in Hep3B cells (Fig. 2C and D). In addition, compared with the control group, DHM and NDP treatment reduced the invasive ability of Hep3B cells (Fig. 2C and D). These results suggested that DHM inhibited cell migration and invasion of Hep3B cells. Notably, the inhibition of migration and invasion in Hep3B cells following DHM treatment was superior to that by NDP treatment.

### DHM induced apoptosis in Hep3B cells

**Fig. 3:**
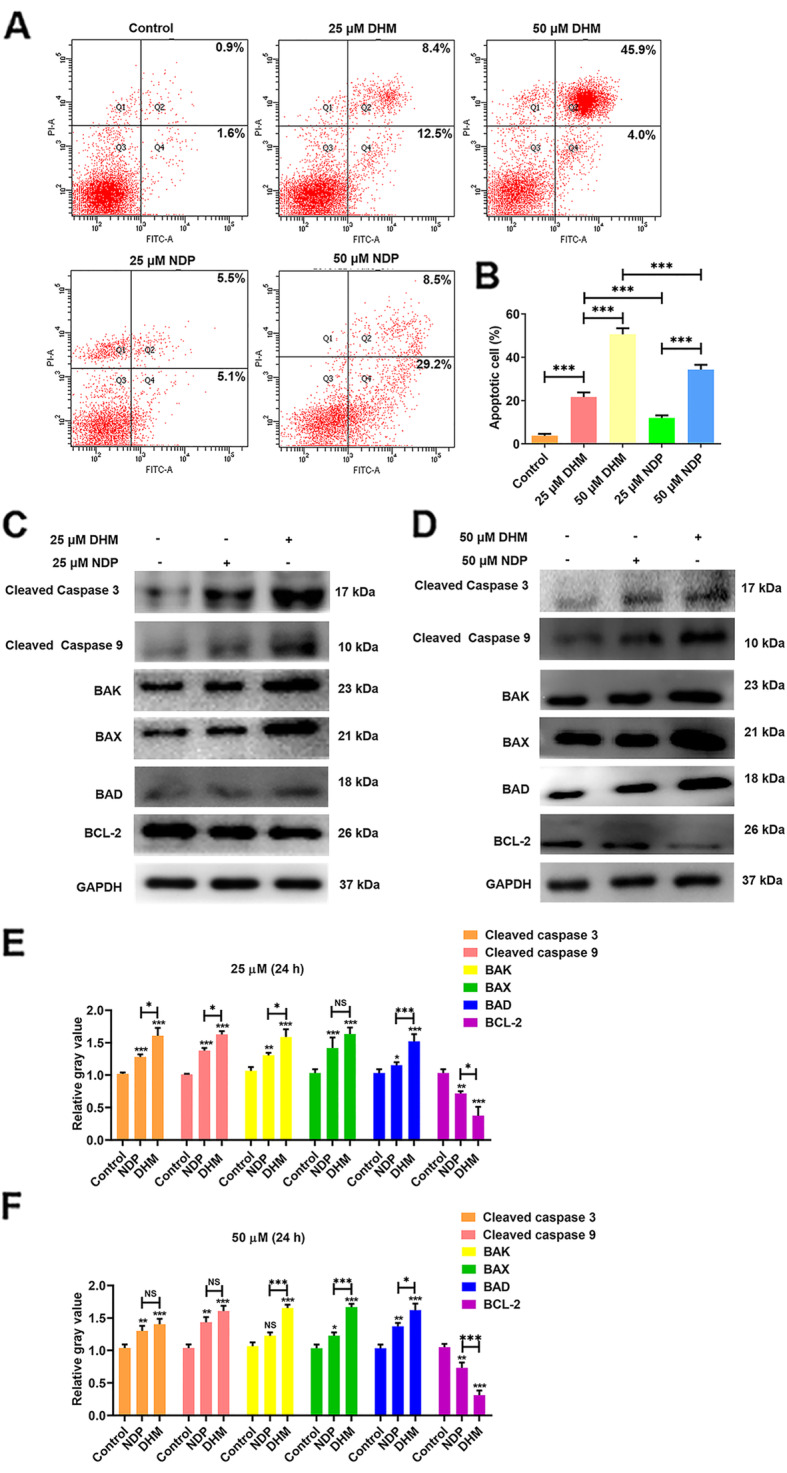
DHM induced apoptosis in Hep3B cells. **A** Following treatment with five different group; Control, DHM (25 and 50 μM) and NDP (25 and 50 μM) for 24 h, apoptosis rate of Hep3B cells was determined using Annexin V-FITC/PI dual-staining flow cytometry. **B** Quantification of apoptosis rate in Hep3B cells detected by Annexin V-FITC/PI dual-staining flow cytometry. **C** and **D** Apoptosis-associated factors (cleaved caspase 3, cleaved caspase 9, caspase 9, BAK, BAX, BAD and BCL-2) were examined by western blot. Results are presented as mean ± standard error of mean (*n* ≥ 3). **E** and **F** Integrated density data were quantified. All images are representative of three independent experiments. NDP, Nedaplatin; PI, propidium iodide. Data are means ± SEM of three independent experiments, ***P < 0.001 vs. control group or NDP group

As shown in Fig. 3A and B, the Hep3B cells were stained with FITC-Annexin-V and PI, and early and late apoptotic cells were measured by flow cytometry. The apoptosis rates of DHM and NDP group were significantly higher than the control group (*P* < 0.001; Fig. 3B), and the apoptosis rate of the DHM group was also significantly higher than the NDP group (*P* < 0.001; Fig. 3A and B). The proportion of apoptotic cells increased from 3.73 ± 1.57% in the control group to 21.7 ± 3.57% in the DHM (25 μM), 12.03 ± 1.98% in the NDP (25 μM), 50.67 ± 4.80% in the DHM (50 μM) and 34.33 ± 3.81% in the NDP (50 μM) experimental groups following treatment for 24 h (Fig. 3B; *P* < 0.001 versus control or NDP group). At 24 h, apoptosis was higher (*P* < 0.001) in a dose-dependent manner of DHM-exposed Hep3B cells compared to those in NDP group. The percentages of early and late apoptotic cells significantly increased with an increase in drug concentration. Thus, DHM induced apoptosis of the Hep3B cells (*P* < 0.001), and the ability of DHM to induce the apoptosis of Hep3B cells was better than NDP. Next, the protein levels of apoptosis-associated factors were measured by western blot. As displayed in Fig. 3C-F, DHM and NDP significantly promoted cleaved caspase 3, cleaved caspase 9, BAK, BAX and BAD expressions and inhibited BCL-2 expression compared with control group (*P* < 0.001). These results suggested that DHM could induce apoptosis in Hep3B cells.

### Anticancer effects of DHM on cancer development in vivo

**Fig. 4:**
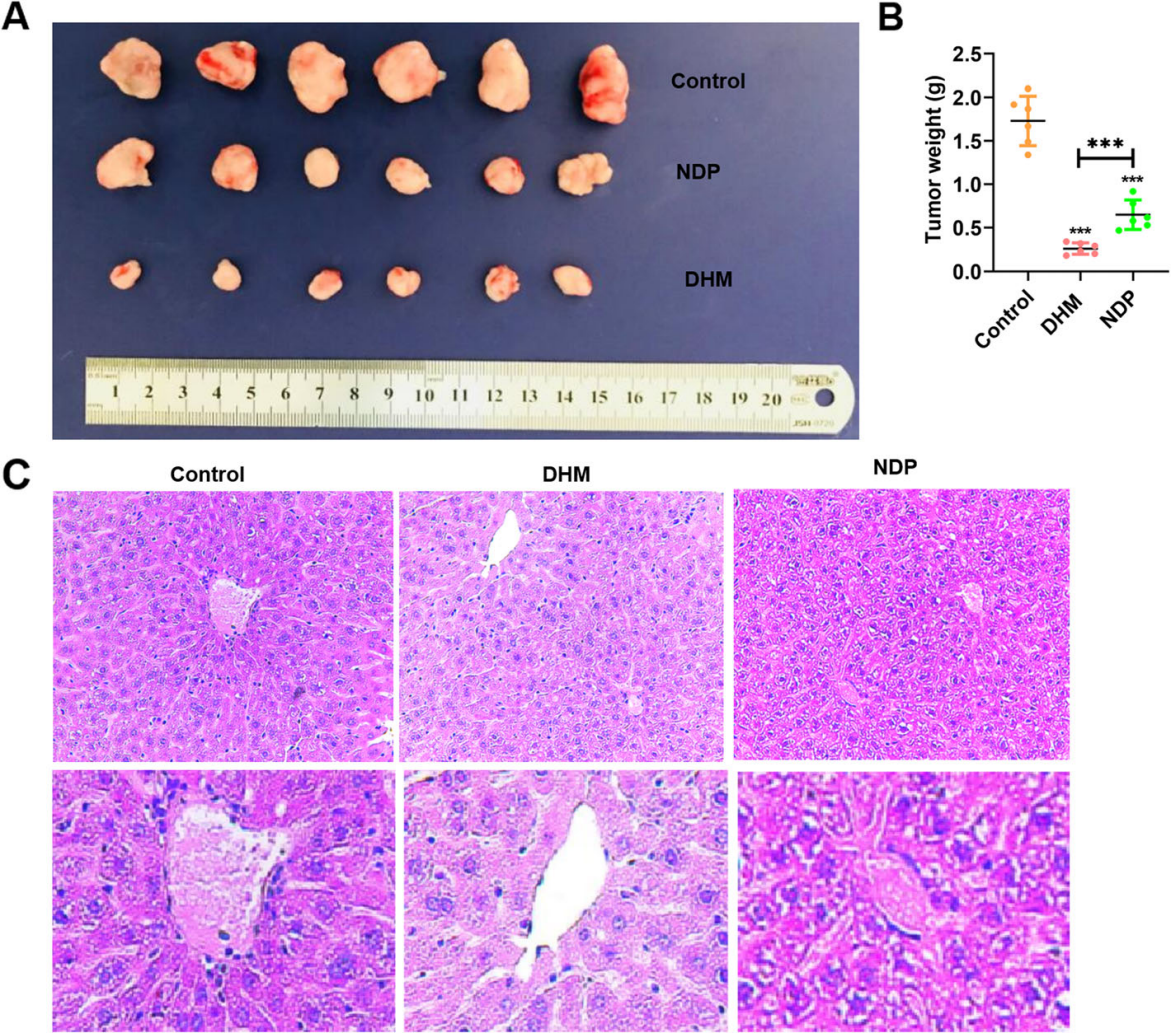
DHM inhibited cancer development in nude mice. **A** After nude mice were sacrificed, they were dissected to obtain tumors and photograph (lower). **B** Excised tumors were weighed separately. **C** Histologic analysis of HE staining of hepatic cords disordered and hepatic sinuses. Values are presented as the mean ± SEM (*n* = 6). *P < 0.05, **P < 0.01 and ***P < 0.001 compared with control or NDP group

The effect of DHM on the growth of primary tumor xenografts in nude mice was examined. Tumor volumes were recorded every three days. The volumes of the primary tumors in the DHM and NDP groups were greatly reduced compared with the control group, and the effect of the DHM treatment was superior to the effect of NDP (Fig. 4A). The weight of the tumors in the DHM group was only 0.26 ± 0.066 g at the end of the experiment compared with the NDP group (0.65 g ±0.169 g) and control group (1.73 ± 0.284 g) (Fig. 4B, *P* < 0.001). These results illustrated that DHM exhibited the inhibition of cancer development. Liver tissues of nude mice were stained with HE (Fig. 4C). Hepatocytes in the DHM group were significantly enlarged, and could confirm the structure of hepatic lobules, which resulted in hepatic cords disordered and hepatic sinuses narrowed (Fig. 4C). Thus, DHM exhibited a better curative effect than NDP in suppressing the cancer development.

## Discussion

At present, the treatment options of HCC mainly include orthotopic liver transplantation, surgical resection, local destruction, radiotherapy, and chemotherapy. Although there have been advances in the treatment of HCC patients, the worldwide recurrence and mortality rates of HCC and HCC-associated cases are very high. NDP is a broad-spectrum anticancer drug, and it may be used in the treatment of malignant tumors, such as cervical, nasopharyngeal, esophageal, and lung cancer [25–29]. In recent years, a number of studies have shown the molecular mechanism of NDP in cancers, and the NDP potentially involved multiple potential mechanisms. However, NDP treatment led to autophagosome accumulation and increased LC3-II expression in cisplatin-sensitive nasopharyngeal cancer cell lines [30]. Furthermore, it has been demonstrated that high concentration of NDP could cause treatment-related side effects, such as nephrotoxicity, hematological toxicity, ototoxicity [2, 31, 32].

DHM, a naturally flavonoids of medicinal plants, has demonstrated therapeutic efficacy in the treatment of various cancer, and it has attracted attention as an anticancer agent against lung cancer, gastric cancer, ovarian cancer and liver cancer. DHM may be combined with or replace other chemotherapeutic drugs, such as NDP, in cancer therapy. Studying these molecular targets also provides novel theoretical foundation for understanding the molecular mechanisms of cancer, as well as novel drugs to replace NDP for cancer treatment. The safety of DHM has been studied in cell cultures, animals, healthy individuals and patients [33, 34], and DHM is generally recognized as a safe extract of Rattan tea [35, 36]. In Hep3B cell culture studies, DHM inhibited cell proliferation and viability, migration, invasion, and promoted apoptosis. Furthermore, DHM treatment inhibited growth of xenotransplanted tumors in mice [37], suggesting the potential therapeutic effects of DHM as an anticancer agent.

The main anticancer mechanisms of DHM that have been described thus far are as follows: inhibition of cell proliferation; induced cell cycle arrest; induces apoptosis. DHM treatment (2, 10, 50, 100 and 200 μM) for 48 h inhibited cell proliferation and induced G2/M phase arrest in HepG2 and Hep3B cells [38]. However, in this study, DHM treatment (25 and 50 μM) for 24 h significantly promoted pro-apoptotic protein expressions, such as cleaved caspase 3, cleaved caspase 9, BAK, BAX and BAD, but inhibited Bcl-2 expression, induced cell apoptosis of Hep3B cells. In addition, activation of the cancer suppressor gene p53 [10], and inhibition of Semaphorin 4D (Sema4D) [39], multidrug resistance protein 2 (MRP2) [40], NF-κB [41], and Notch1 pathway [14] and angiogenesis, can promote apoptosis and cytoprotective autophagy [41]. However, to the best of our knowledge, the anticancer effect of DHM in Hep3B cells has rarely been reported to date. The purpose of this study was to determine the anticancer effects of DHM on the proliferation, migration and apoptosis of Hep3B cells, implying that DHM may serve as a promising bioactive component for HCC treatment.

As is well known, caspases regulate cell proliferation and apoptosis [42]. Caspase family are usually divided into three protein categories: apoptosis initiators (caspase-9), apoptosis executioners (caspase-3 and -7) and inflammation mediators [43]. Previous research showed that caspase-3 and caspase-9, are key apoptosis proteins in the apoptosis pathway [44]. The caspase-9 protein is the apoptosis initiator and the apoptosis executors (caspase-3 and -7) of cell apoptosis in mammals. The apoptosis initiator is first activated by apoptosis signals, followed by activation of apoptosis executioners of the downstream cascade. Ultimately, large amounts of substrates in cells are hydrolyzed for disintegration. Caspase-3 and caspase-9 are situated at vital junctions in apoptotic signaling pathways. Western blot analyses indicated that DHM treatment markedly promoted cleaved caspase 3, cleaved caspase 9, BAK, BAX and BAD expressions, while inhibited BCL-2 expression in Hep3B cells, which were consistent with other apoptosis-related experiments in human myelomonocytic lymphoma cells [16]. Notably, DHM treatment matched or even exceeded the effect of NDP treatment on the caspase expression levels in Hep3B cells.

Invasion and metastasis are key biological characteristics of malignant cancers. Adhesion molecules are involved in its malignant progression, invasion and metastasis. Cancer cells can invade stromal tissue the host stromal of the target organ by the blood vessel wall, which subsequently promotes cancer metastasis and invasion [45]. Transwell assays confirmed that DHM inhibited the migration and invasion of Hep3B cells in a dose-dependent manner, which was consistent with the results of Chen et al. reported that DHM reduced human cholangiocarcinoma cells migration and invasion [13]. Moreover, DHM treatment significantly reduced the weight of the HCC cancers. Thus, the data showed that the anticancer effects of DHM was better than that of NDP treatment.

## Conclusion

These data demonstrated that DHM inhibited cell proliferation, migration, invasion, and promoted apoptosis of Hep3B cells. DHM may be critical for cell apoptosis and metastasis. The study hinted that DHM exhibited the anticancer effect on HCC, and might provide a novel sight into the clinical treatment of HCC. Further studies are still needed to uncover more potential effect of DHM on HCC.

## Supplementary Information

**Additional file 1.**

## Data Availability

All data and materials used in this current study are available from the corresponding author on reasonable request.

## Abbreviations

ANOVA: One-way analysis of variance
CCK-8: Cell Counting Kit-8
DHM: Dihydromyricetin
HCC: Hepatocellular carcinoma
TCMs: Traditional Chinese medicines
DMEM: Dulbecco’s modified Eagle medium
DMSO: Dimethylsulfoxide
NDP: Nedaplatin
FBS: Fetal bovine serum
