# Supplementary figures and images for: Anticancer effects of dihydromyricetin on the proliferation, migration, apoptosis and in vivo tumorigenicity of human hepatocellular carcinoma Hep3B cells
Source: BMC Complement Med Ther. 2021 Jul 6;21:194. doi: 10.1186/s12906-021-03356-5 (PMC8258952; doi:10.1186/s12906-021-03356-5)

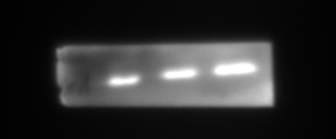

Supplement: Supplementary file 1 — Additional file 1. [file 12906_2021_3356_MOESM1_ESM.zip › BAD.tif]

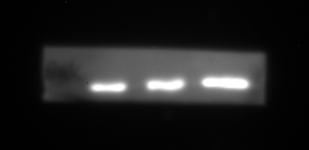

Supplement: Supplementary file 1 — Additional file 1. [file 12906_2021_3356_MOESM1_ESM.zip › BAK.tif]

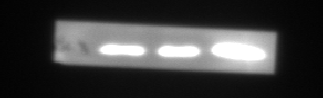

Supplement: Supplementary file 1 — Additional file 1. [file 12906_2021_3356_MOESM1_ESM.zip › BAX.tif]

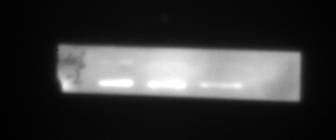

Supplement: Supplementary file 1 — Additional file 1. [file 12906_2021_3356_MOESM1_ESM.zip › BCL2.tif]

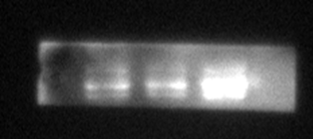

Supplement: Supplementary file 1 — Additional file 1. [file 12906_2021_3356_MOESM1_ESM.zip › Cleaved caspase 3.tif]

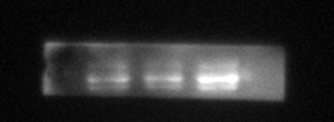

Supplement: Supplementary file 1 — Additional file 1. [file 12906_2021_3356_MOESM1_ESM.zip › Cleaved caspase 9.tif]

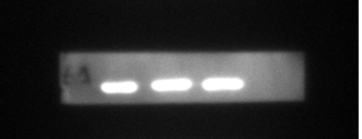

Supplement: Supplementary file 1 — Additional file 1. [file 12906_2021_3356_MOESM1_ESM.zip › GAPDH.tif]
